# ASSOCIATION BETWEEN SENSORY IMPAIRMENTS AND PARTICIPATION IN COMMUNITY ACTIVITIES AMONG OLDER ADULTS

**DOI:** 10.1093/geroni/igae098.2526

**Published:** 2024-12-31

**Authors:** Zoe Shannon, Bernard A Steinman

**Affiliations:** University of Wyoming, Laramie, Wyoming, United States; University of Wyoming, Laramie, Wyoming, United States

## Abstract

Older adults with sensory impairments (SI) experience barriers to participation that could impact their quality of life and well-being. Participation limitations associated with SI have also been associated with health-related outcomes, including chronic disease, mobility difficulties, impairments in daily activities, and demographic factors. In this study, we assessed the association between functional SI and participation in community activities, while holding other health dimensions constant. Using two waves (2011 and 2015) of the National Health and Aging Trends Study (NHATS), we computed five ordinal logistic regression models to assess associations between SI change and participation. Sensory impairments were operationalized via self-reported measures of everyday visual, hearing, and dual functioning (e.g., ability to carry on a conversation in a quiet room). “Participation” was operationalized using six items that assessed participation in social and community-based activities (e.g., doing volunteer work). We created a change-in-participation variable by subtracting participation in 2015 from the baseline. Without other covariables, only functional vision status was statistically associated with participation. When all other covariates were included in the final model, statistical effects of functional vision were eliminated, and only age and chronic health conditions predicted change in participation. These results point to the importance of developing community support and reducing barriers to participation by older adults with functional SI. Creating social and physical environments that consider vision, hearing, and dual sensory impairments can foster greater participation among older adults with SI. Consideration of sensory functioning can also promote well-being that is associated with continued engagement in the community.

